# Effects of pepper – peanut intercropping systems on processed chili yield and rhizospheric soil microecological environment

**DOI:** 10.3389/fpls.2025.1666686

**Published:** 2025-10-14

**Authors:** Jingjing Wang, Xiapu Gai, Youping Wang, Peipei Chang, Tengfei Li, Shaoli Zhang, Hongyong Zhang, Hongbo Li, Zikun Zhang

**Affiliations:** ^1^ Dezhou Academy of Agricultural Sciences, Dezhou, China; ^2^ Institute of Pepper, Guizhou Academy of Agricultural Sciences, Guiyang, China

**Keywords:** pepper – peanut, intercropping mode, photosynthetic index, soil nutrients, soil enzyme activity, soil microorganisms, production

## Abstract

**Introduction:**

This study investigated the effects of pepper - peanut intercropping patterns on the rhizosphere soil microenvironment and yield of processing chili.

**Methods:**

Using the processing chili varieties “*Beike 802*” and “*Dehong 1*” as test materials, treatments included monoculture (BK, DH) and peanut intercropping (BKIM, DHIM). The dry matter accumulation, agronomic traits, photosynthetic parameters, soil nutrients, enzyme activities and microbial community changes were analyzed by split plot experiment design.

**Results and discussion:**

The results demonstrated that intercropping significantly enhanced dry matter accumulation in pepper plants (the dry matter accumulation of pepper was increased by 25.25% in BKIM compared with BK in full fruit period, p<0.05). Yield per 667 m^2^ increased by 9.12% to 15.01%, and the number of fruits per plant rose by 10.14% to 13.39%, with differences being statistically significant (*p* < 0.05). Photosynthetic parameters—including net photosynthetic rate (Pn), stomatal conductance (Gs), and transpiration rate (Tr)—were significantly higher under intercropping at the full fruit stage (*p* < 0.05), while intercellular CO_2_ concentration also increased synchronously. Soil nutrient analysis revealed that intercropping significantly increased organic matter (e.g., DHIM reached 19.92 g kg^-^¹) and available phosphorus content but reduced available potassium levels (*p* < 0.05). Microbial community analysis indicated a significant rise in bacterial and fungal operational taxonomic units (OTUs) under intercropping (e.g., bacterial OTUs in BKIM increased by 91.26% compared to BK, *p* < 0.01). The abundance of key beneficial taxa such as Proteobacteria and Chytridiomycota was enhanced, and soil microbial diversity indices (ACE and Chao1) were markedly higher in intercropped treatments (*p* < 0.05).

**Conclusion:**

In summary, pepper - peanut intercropping significantly promoted pepper yield by optimizing photosynthetic efficiency and improving soil microecology, providing a theoretical basis for alleviating continuous cropping obstacles.

## 1 Introduction

*Capsicum annuum L* is an herbaceous plant in the genus Capsicum and it has become the most extensively cultivated vegetable in China, with its sown area consistently exceeding 2.1 million hectares in recent years (Han et al., 2024). Processing chili refers to a type of chili pepper specifically cultivated for food processing, condiment production, and the manufacture of chili pepper products, such as chili powder, chili sauce, dried chili peppers, chili oil, chili extract, etc. These peppers are not as fresh vegetables directly but are supplied as raw materials to food industry. The processing chili industry has developed rapidly, and its planting area accounts for about 50% of the pepper planting area (Zou et al., 2020). The processing of chili peppers has high economic added value, including the extraction of high-value components such as capsaicin, diversified applications in the fields of medicine and food, and exports. Selecting and breeding varieties suitable for processing chili peppers is of great significance for adapting to the needs of the processing market and promoting the upgrading of the chili pepper industry. As an important condiment and industrial raw material, processed chili is in great demand at home and abroad (Wang et al., 2021). Shandong Province cultivates approximately 120,000 hectares of pepper, with processing chili varieties accounting for 80% of the total area. It has become a vital raw material production base for processing chili in China., Meanwhile, the region has evolved into both a trading hub for processing chilis in northern China and a modern agricultural technology radiation zone specializing in primary and deep processing stages, with key hubs in Dezhou city, Qingdao city and Jining city.

The single planting of *Capsicum annuum L* is prone to exacerbation of soil-borne diseases, resulting in adverse symptoms such as dwarfed plants and wilted leaves and poor economic benefits. Especially with the continuous expansion of pepper planting scale, the continuous cropping obstacle was serious, the resistance of varieties decreases, the yield was low, and the fruit quality was poor. It has become an important factor restricting the sustainable development of pepper. The intercropping model is a common agricultural tillage method, which improves system productivity through resource complementation (light, water, nutrients) and biological interactions (Weng et al., 2021), reduces the occurrence of pests and diseases, and thus improves crop yield and economic benefits (Zhao et al., 2001; Gao et al., 2017), and its yield-increasing effect has been confirmed in grass - legume models (such as maize - soybean) (Brooker et al., 2015). Intercropping patterns of chili peppers with corn, soybeans, leguminous crops, and peanuts have been successfully practiced globally and in various regions of China (Xiao et al., 2003b; Wang et al., 2019). *Arachis hypogaea L* is a legume crop. Its root symbiotic nitrogen fixation system could fix nitrogen 40–200 kg N ha^-1^ per year (Peoples et al., 2009), and the root secreted organic acids (such as malic acid, citric acid) to activate soil insoluble phosphorus (Hinsinger et al., 2003). Previous studies have shown that sugarcane - peanut intercropping could significantly improve the soil nutrients and soil enzyme activities of crops (Qiu et al., 2024), maize - peanut intercropping could improve the photosynthetic rate of crops (Chen et al., 2023). Dai et al. (2015) also found that tomato intercropping legumes could have a better soil fertility effect, thereby significantly increasing yield. The choice of intercropping peanuts with chili peppers is based on the consideration that the two crops can achieve complementarity in ecological niches and resource utilization, improve the field microenvironment, achieve synergistic effects in pest and disease control and enhance economic benefits.

The essence of continuous cropping obstacles in *Capsicum annuum L* lies in the imbalance of the “root exudates-soil microbiome-nutrient cycling” triad: (1) Accumulation of phenolic allelochemicals suppressing root growth (Wu et al., 2015); (2) The abundance of beneficial bacteria such as Actinobacteria decreased, while pathogens such as Fusarium were enriched (Zhou et al., 2019); (3) The availability of phosphorus and potassium decreased in alkaline soil (pH > 8.0) (pH 8.44). Legume intercropping could alleviate obstacles through three mechanisms: biological nitrogen fixation to supplement nitrogen sources (Jensen et al., 2020), root interactions reshaping microbial communities (Li et al., 2016) and canopy modulation improving light-thermal microenvironments (Gao et al., 2010). However, in the Huang-Huai-Hai Plain (Dezhou is a typical area of the experimental site), pepper - peanut intercropping mode was spontaneously adopted by farmers, but there was a lack of systematic analysis of its micro-ecological mechanism.

High-throughput sequencing technology reveals the “cry for help” mechanism in the rhizosphere microbiome: Stress-affected crops recruit beneficial microbes to counteract adversity (Berendsen et al., 2018). In intercropping systems, this recruitment may occur through cross-species root-root dialogue: Peanut roots secrete flavonoids that activate PGPR (Plant Growth-Promoting Rhizobacteria) in the pepper rhizosphere, such as *Pseudomonas* spp (Vandenkoornhuyse et al., 2015). However, current research exhibits three critical limitations:(1) Overemphasis on bacterial communities while overlooking fungal roles (particularly functional groups like *Chytridiomycota*); (2) Lack of dynamic monitoring across developmental stages (from initial flowering → maturity); (3) A research void exists regarding the micro-ecology of intercropping systems in alkaline soil environments.

Based on the aforementioned background, this study proposes a core hypothesis: Pepper - peanut intercropping enhances pepper yield by remodeling the rhizosphere microbial network, thereby driving soil nutrient activation and photosynthetic efficiency improvement. To test this hypothesis, we implemented a split-plot design (main plot: pepper cultivar; subplot: cropping pattern) and conducted dynamic monitoring across three critical growth stages of pepper: Plant responses: dry matter accumulation , photosynthetic parameters (Pn, Gs, Tr, Ci), yield components; Soil environment: nutrient dynamics (N, P, K), enzyme activities (urease, sucrase, etc.); Microbiome profiling: 16S rRNA + ITS high-throughput sequencing (OTUs, diversity indices, phylum-level abundance) index change, to clarify the effect of intercropping with peanut on the rhizosphere soil microenvironment and yield of processing chili, provide a theoretical basis for further optimization and promotion of pepper - peanut intercropping planting mode, and is of great significance to the sustainable development of processing chili industry.

## 2 Materials and methods

### 2.1 Test materials

The processing chili test material *“Dehong 1”* was bred by Dezhou Academy of Agricultural Sciences, and *“Beike 802”* was provided by Henan Beike Seed Industry Co., Ltd. The peanut test material was *“Luhua No.1”*, which was purchased from Tianqu New District of Dezhou City as the agricultural service center.

### 2.2 Test site conditions

The experiment was carried out in the modern agricultural science and technology park of Dezhou Academy of Agricultural Sciences. The soil texture is loam, the terrain is flat, and the irrigation and drainage are convenient. The topsoil contains total nitrogen 0.91 g kg^-1^, available phosphorus 51.46 mg kg^-1^, available potassium 176.43 mg kg^-1^, organic matter 17.04 g kg^-1^, and the pH value is 8.44.

### 2.3 Experimental design

The experiment was carried out in 2023.In early March, the floating seedling technology was used to raise seedlings. It was planted in early May. The split plot experiment design was adopted, the variety was the main area, and the planting mode was the sub-area. Four treatments were set up: *Beike 802* and peanut intercropping (BKIM), *Beike 802* monoculture (BK), *Dehong 1* and peanut intercropping (DHIM) and *Dehong 1* monoculture (DH), each treatment repeated three times.

**Figure 1:**
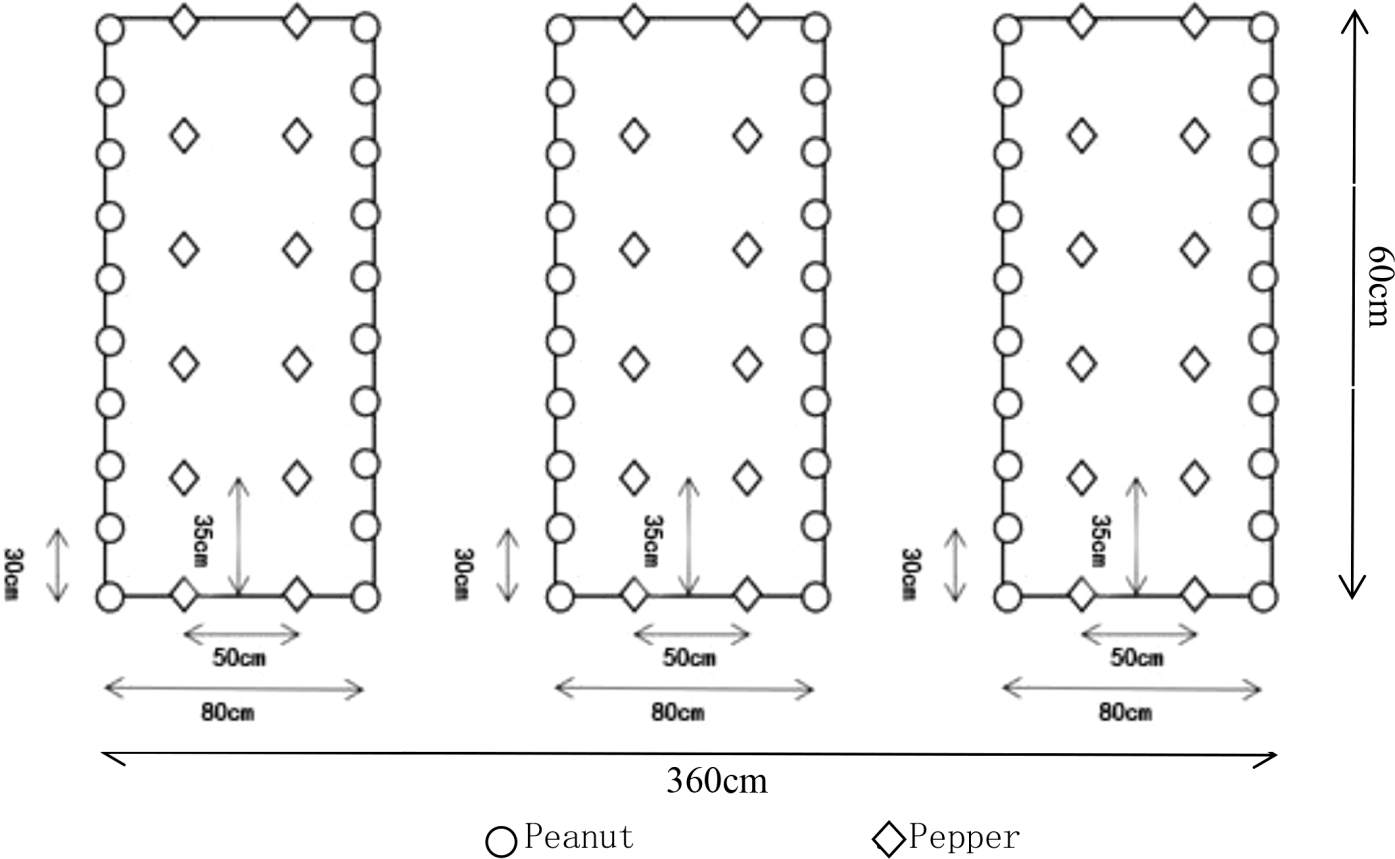
Peanut pepper intercropping plot diagram.

The planting of pepper was carried out according to the 140 cm pull line, the width of the ditch was 60 cm, the width of the border was 80 cm, and the length was 6 m. Three borders were a plot, and the planting area of each plot was 21.6 m^2^. 6–8 leaves of seedling age with relatively consistent growth were selected to start transplanting. One plant per hole. Pepper was harvested in mid-August. Pepper and peanut were intercropped according to 2:2, that is, 2 rows of pepper and 2 rows of peanut, 2 rows of pepper were planted in the middle of each border, and peanuts were planted on both sides of the border (interlaced with pepper). The plant spacing of peanut was 30 cm, and the plant spacing of pepper was 50 cm × 35 cm. The planting density was 2565 plants per 667 m^2^ for *Dehong 1* and 6670 plants per 667 m^2^ for *Beike 802*. Pepper was planted in two rows in the middle of each border, and the row spacing was 50 cm × 35cm. The planting density was consistent with pepper intercropping. See **Figure 1** below for details. In addition to the different planting modules, other field management measures were carried out in accordance with the habits of local farmers and remained consistent.

### 2.4 Sampling period and soil sampling method

Samples were taken at the early flowering stage, full fruit stage and mature stage of pepper, and 3 plants were selected from each treatment to take 300 g of rhizosphere soil. The whole root system of pepper was dug out from the soil by shaking soil method (Lu et al., 2015), and the soil around the root system which was loosely connected with the root system was taken. The impurities such as stones and plant residual roots were picked out, dried and sieved, and the soil enzyme activity and nutrient were determined.

### 2.5 Determination of indicators and methods

#### 2.5.1 Determination of soil nutrients

The content of alkali-hydrolyzable nitrogen was determined by alkali solution diffusion method, the content of available phosphorus was determined by molybdenum antimony colorimetric method, the content of available potassium was determined by NH_4_OAc extraction flame photometer, and the pH value was determined by acidity meter and potential method.

#### 2.5.2 Determination of soil enzyme activity

Soil urease activity, soil sucrase activity, soil acid phosphatase and soil catalase activity were determined by sodium phenol-sodium hypochlorite colorimetry, 3,5-dinitrosalicylic acid colorimetry, disodium phenyl phosphate colorimetry and potassium permanganate titration, respectively.

#### 2.5.3 DNA extraction, PCR amplification and high-throughput sequencing

The total DNA of soil samples was extracted by soil DNA extraction kit, and its concentration and purity were detected by 0.8% agarose gel electrophoresis and ultramicro nucleic acid spectrophotometer. The V3 − V4 hypervariable region of the 16S rRNA gene sequence was amplified using bacterial universal primers 338F (5’-ACTCCTACGGGAGGCAGCA-3’) and 806R (5’-GG ACTACHVGGGTWTCTAAT-3’). The fungal ITS1 − ITS2 region was amplified by fungal universal primers ITS1F (5’-CTTGGTCATTTAGAGGAAGTAA-3’) and ITS2 (5’-GCTGCGTTCTTCATCGATGC-3’). After the PCR amplification product was detected by electrophoresis, the target fragment was recovered using a gel recovery kit. The recovered products were sent to the sequencing company for subsequent high-throughput sequencing and sequence analysis.

#### 2.5.4 Agronomic traits and yield determination

Three representative plants were taken from each plot to determine the agronomic traits of pepper, and the yield was measured after harvest.

In this paper, the drying method is used to determine the dry matter content of chili peppers. Dry matter content refers to the percentage of dry matter in the sample relative to its fresh weight. The calculation formula is: Dry matter content (%) = (Dry weight/Fresh weight) × 100%.

#### 2.5.5 Determination of net photosynthetic rate (Pn), stomatal conductance (Gs), transpiration rate (Tr) and intercellular CO_2_ concentration (Ci) in leaves

The photosynthetic parameters were measured at the early flowering stage of pepper, once every 15 days for 4 times. Using Li-6800 portable photosynthesis system (Li-COR company) at 9:00 – 11:00 am, three representative plants were randomly selected from each plot, and the photosynthetic indexes (Pn, Gs, Tr, Ci) of the top three leaves of the main stem were measured.

### 2.6 Data analysis

Excel 2013 and DPS v7.05 software were used for data analysis and mapping, and Duncan’s test was used for multiple comparisons of significant differences (*P* < 0.05).

## 3 Results

### 3.1 Effects of pepper - peanut intercropping on dry matter accumulation

At different growth stages, the aboveground, underground and total dry matter accumulation of the two peppers showed an increasing trend (**Table 1**), and the intercropping treatment (BKIM, DHIM) was higher than that of monoculture (BK, DH). From full fruit stage to mature stage, the dry matter accumulation of BKIM and DHIM were 211.47 g, 295.63 g and 376.88 g, 422.03 g, respectively, which were significantly increased by 25.25%, 21.11% and 17.94%, 15.65%, respectively, compared with BK and DH. The total dry matter accumulation of BKIM and DHIM were 233.75 g, 325.26 g and 391.30 g, 439.62 g, respectively, which were significantly increased by 25.46%, 22.72% and 18.02%, 15.62%, respectively, compared with BK and DH. During the full fruiting period, the plants bloom profusely, and the fruits grow rapidly and continuously, with an increasing trend in dry matter accumulation. By the mature stage, the fruit peel continues to develop, changing color from green to red, and the dry matter content reaches its maximum. It shows that the pepper - peanut intercropping mode is beneficial to the accumulation of dry matter of pepper.

**Table 1:** Changes of dry matter accumulation of pepper under pepper - peanut intercropping mode.

Dry matter accumulation in peppers	Treatment	BKIM	BK	DHIM	DH
Underground portion (g sytrain^-1^)	Initial flowering	7.67a	6.24a	9.15a	8.88a
	Peak Fruiting Stage	22.28a	17.48a	14.42b	12.00b
	Maturity	29.63a	20.93b	17.59c	15.30c
Overground part (g sytrain^-1^)	Initial flowering	61.18b	55.03b	128.02a	109.80a
	Peak Fruiting Stage	211.47c	168.84d	376.88a	319.56b
	Maturity	295.63c	244.11d	422.03a	364.92b
Total dry matter accumulation (g sytrain^-1^)	Initial flowering	68.85c	61.27c	137.17a	118.68b
	Peak Fruiting Stage	233.75c	186.32d	391.30a	331.56b
	Maturity	325.26c	265.04d	439.62a	380.22b

Different lowercase letters represent significant difference level (*P* < 0.05).

### 3.2 Effects of pepper - peanut intercropping on agronomic traits and yield of pepper

From **Table 2** it is evident that the intercropping mode (BKIM, DHIM) is superior to the monoculture treatment (BK, DH). In terms of yield components, the yield per 667 m^2^ of BKIM and DHIM was 20100.64 kg and 2601.71 kg, respectively, which was 15.01% and 9.12% higher than that of monoculture, and the difference was significant. The fruit number per plant of BKIM and DHIM were significantly increased by 13.39% and 10.14%, respectively, compared with monoculture. The fruit length, fruit width, single fruit weight and yield per plant of BKIM and DHIM were also higher than those of monoculture, but the difference was not obvious. In terms of agronomic traits, the plant height, plant width and stem diameter of BKIM and DHIM were also slightly higher than those of monoculture. It shows that the pepper - peanut intercropping mode is beneficial to the growth of pepper.

**Table 2:** Changes of agronomic traits and yield of pepper under pepper - peanut intercropping mode.

Treatment	BKIM	BK	DHIM	DH
Plant height (cm)	104.04a	100.21a	106.6a	103.35a
Plant width (cm)	54.10b	52.73b	66.42a	63.5a
Stem thick (cm)	2.16a	1.98a	2.76a	2.37a
Fruit length (cm)	6.10b	5.90b	16.15a	15.91a
Cone width (mm)	1.35b	1.30b	2.14a	1.82a
Fruit flesh thickness (mm)	1.61b	1.48b	2.19a	2.04a
Number of fruit per plant (number)	184.60a	162.80b	115.32c	104.70d
Number of red fruits per plant (number)	110.00a	96.60b	66.50c	64.00c
Fruit weight (g)	2.32b	2.29b	9.70a	9.63a
Yield per tree (kg)	0.36b	0.31b	0.93a	0.85a
Yield per 667m^2^ (kg)	2010.64c	1748.17d	2601.71a	2384.16b

Different lowercase letters represent significant difference level (*P* < 0.05).

### 3.3 Effects of pepper - peanut intercropping on photosynthetic indexes of pepper

**Figure 2:**
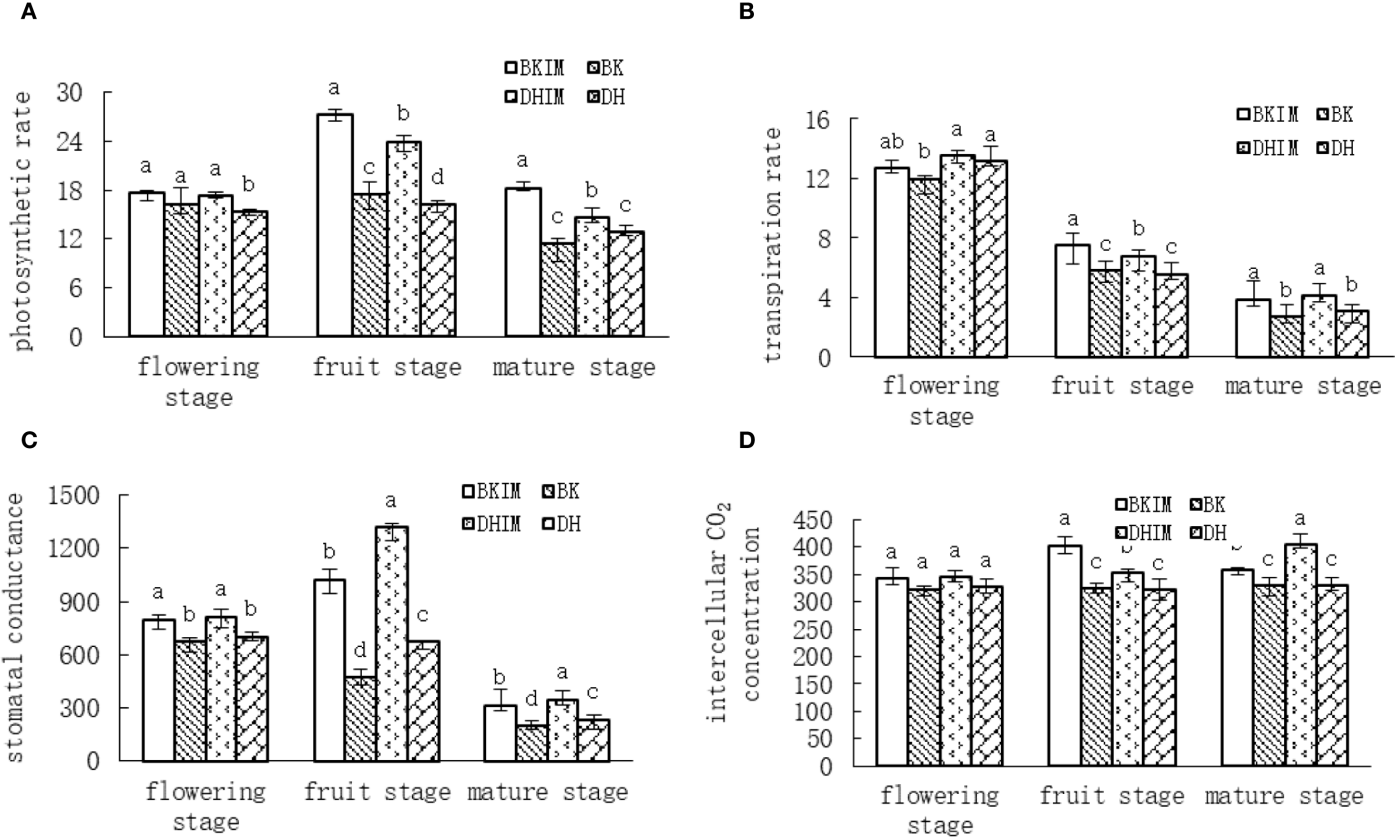
Changes of photosynthetic indexes of pepper under pepper - peanut intercropping mode. **(A)** represents Photosynthetic rate Pn changes. **(B)** represents Tr change of transpiration rate. **(C)** represents stomatal conductance gs change. **(D)** represents Intercellular CO2 concentration Ci changes. Different lowercase letters represent significant differences (*P* < 0.05).

According to **Figure 2A**, the photosynthetic rate of pepper showed a trend of increasing first and then decreasing. The photosynthetic rate of each treatment was the highest at the full fruit stage, and it was significantly higher than that of monoculture from the full fruit stage to the mature stage. BKIM and DHIM increased by 56.61%, 47.93% and 59.06%, 13.46%, respectively, compared with monoculture BK and DH.

It could be seen from **Figure 2B** that the transpiration rate of pepper under different treatments showed a decreasing trend. From the early flowering stage to the mature stage, BKIM increased by 6.20%, 28.74% and 45.32% respectively compared with BK, and DHIM increased by 3.05%, 21.72% and 35.29% respectively compared with DH.

It could be seen from **Figure 2C** that the change of stomatal conductance of pepper in different periods is similar to that of photosynthetic rate, showing a trend of increasing first and then decreasing. From the early flowering stage to the mature stage, BKIM increased by 18.61%, 116.67% and 58.15% respectively compared with BK, and DHIM increased by 16.05%, 95.56% and 49.77% respectively compared with DH.

It could be seen from **Figure 2D** that at different growth stages of pepper, compared with monoculture (BK, DH), the intercellular CO_2_ concentration under intercropping treatment (BKIM, DHIM) tended to increase. There was no significant difference in the early flowering stage. BKIM and DHIM increased by 24.16% and 9.30% respectively compared with monoculture BK and DH at the full fruit stage, and BKIM and DHIM increased by 8.65% and 22.69% respectively compared with monoculture BK and DH at the mature stage.

### 3.4 Effects of pepper - peanut intercropping on soil nutrients

It could be seen from **Table 3** that with the advancement of pepper growth process, the soil total nitrogen content, available phosphorus and available potassium content of the two peppers showed an overall downward trend, and the total nitrogen content did not change significantly. The available phosphorus was higher than that of monoculture (BK, DH) under intercropping treatment (BKIM, DHIM), and the difference was significant under DHIM treatment. The content of available potassium in intercropping mode (BKIM, DHIM) was significantly lower than that in monoculture (BK, DH), and the content was the highest at the early flowering stage, reaching 267.71 mg kg^-1^. At different growth stages, the content of organic matter increased first and then decreased. The highest content was 19.92 g kg^-1^ in the full fruit period, and the intercropping mode (BKIM, DHIM) was significantly higher than the monoculture (BK, DH). The pH value of rhizosphere soil of pepper in different growth stages did not change significantly, and the pH value of intercropping was lower than that of monoculture, ranging from 8.39 to 8.61.

**Table 3:** Changes of rhizosphere soil nutrient content under pepper - peanut intercropping pattern.

Period	Treatment	Total nitrogen (g kg^-1^)	Available phosphorus (mg kg^-1^)	Available potassium (mg kg^-1^)	Organic matter (gkg^-1^)	pH
Initial flowering	BKIM	1.05a	86.07a	200.64c	16.41b	8.43a
	BK	1.00a	83.50ab	267.71a	17.30ab	8.45a
	DHIM	1.09a	85.57a	198.94d	16.40b	8.41a
	DH	1.00a	81.89b	232.12b	17.75a	8.44a
Peak Fruiting Stage	BKIM	1.03a	58.87bc	183.37c	18.24b	8.39a
	BK	0.99a	53.78c	211.55a	17.58c	8.42a
	DHIM	1.01a	69.52a	180.47d	19.92a	8.46a
	DH	1.00a	63.18b	199.17b	18.06bc	8.52a
Maturity	BKIM	1.01a	52.94b	149.93b	17.38a	8.54a
	BK	0.98a	44.12c	165.92a	16.79b	8.61a
	DHIM	0.99a	61.44a	127.38d	15.80bc	8.46a
	DH	0.98a	44.32c	129.06c	15.40c	8.60a

Different lowercase letters represent significant difference level (*P* < 0.05).

### 3.5 Effects of pepper - peanut intercropping on soil enzyme activities

It could be seen from **Table 4** that with the growth of pepper, the enzyme activity of rhizosphere soil showed a decreasing trend. At the early flowering stage and the full fruit stage, the intercropping treatment (BKIM, DHIM) was lower than the monoculture (BK, DH), and the catalase and urease were significantly different. At the mature stage, the intercropping treatment (BKIM, DHIM) was higher than the monoculture (BK, DH), and the sucrase and urease were significantly different.

**Table 4:** Changes of soil enzyme activity under pepper - peanut intercropping pattern.

Period	Treatment	Sucrase (mg d g^-1^)	Catalase (μmol d g^-1^)	Urease (μg d g^-1^)	Acid phosphatase (μmol d g^-1^)
Initial flowering	BKIM	97.88c	52.40a	570.26b	9.23c
	BK	105.17a	50.35b	581.87a	10.95a
	DHIM	102.57b	52.32a	557.67c	9.52bc
	DH	104.04ab	47.25c	572.03b	9.86b
Peak Fruiting Stage	BKIM	108.24a	47.88c	548.02c	9.73b
	BK	108.56a	46.18d	562.06b	11.46a
	DHIM	106.90b	54.33a	527.36d	7.94c
	DH	106.79b	50.26b	584.90a	9.54b
Maturity	BKIM	83.07a	49.99b	535.42b	10.60a
	BK	80.75b	49.16b	506.83c	6.37c
	DHIM	79.25b	52.48a	552.48a	8.76b
	DH	74.36c	51.76ab	533.85d	7.19bc

Different lowercase letters represent significant difference level (*P* < 0.05).

### 3.6 Effects of pepper - peanut intercropping on soil microbial colonies

The high-throughput sequencing technology was used to sequence the soil fungal ITS sequence and bacterial 16S sequence under the pepper - peanut intercropping mode. After double-end splicing, quality control, chimera filtering, etc., high-quality data statistics were performed. The results are shown in **Table 5**. The number of fungal effective sequences of BKIM and DHIM in intercropping mode was 54087 and 63464, respectively, and the number of bacterial effective sequences was 33404 and 36848, respectively, which was higher than that of monoculture BK and DH.

**Table 5:** Changes of soil effective sequence number under pepper - peanut intercropping pattern.

Treatment	Number of effective sequences	
	Fungi	Bacteria
BKIM	54 087	33 404
BK	49 969	19 737
DHIM	63 464	36 848
DH	57 817	35 750

**Figure 3:**
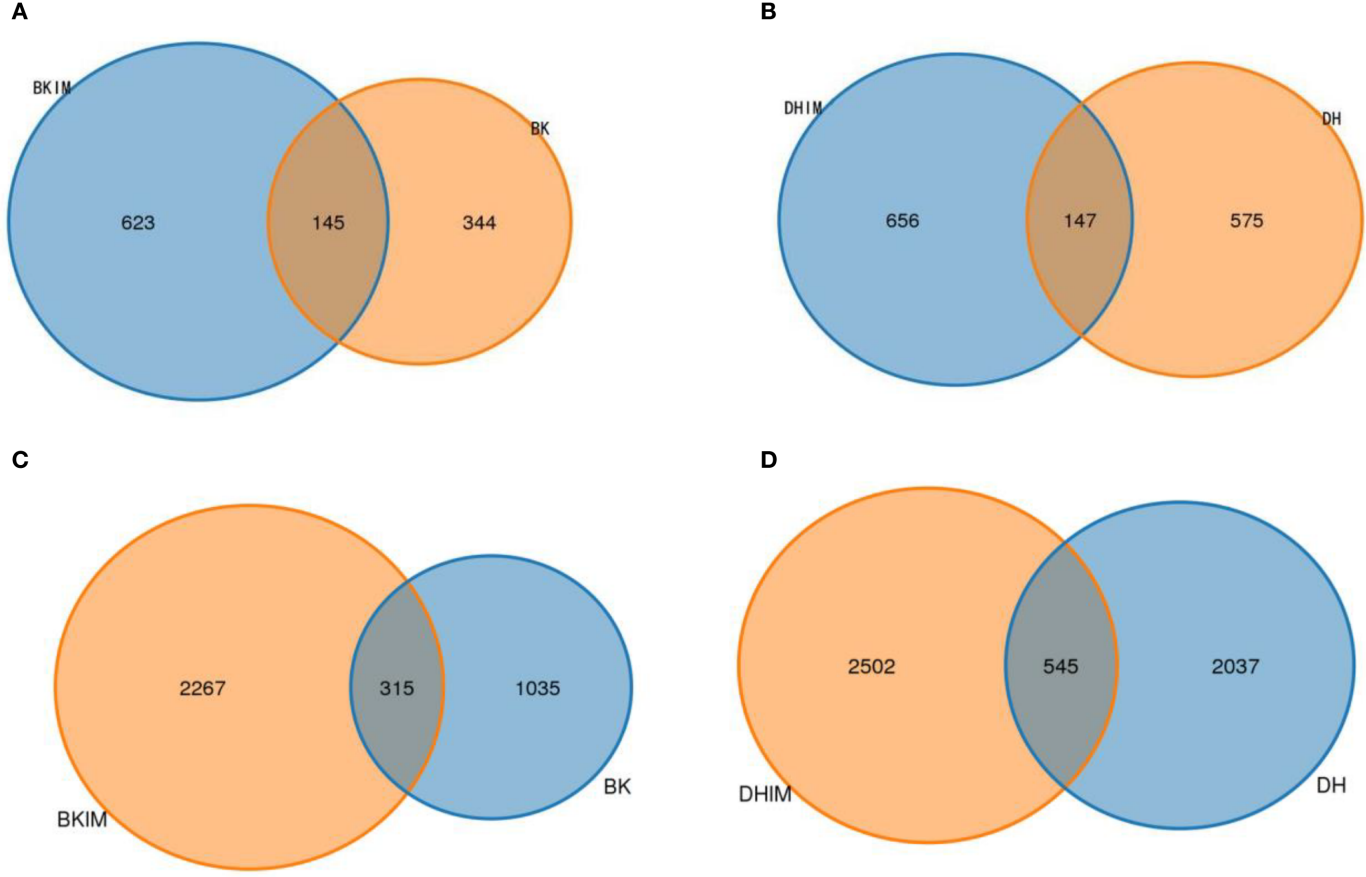
OTU Wayne diagram under pepper - peanut intercropping mode. **(A, B)** represent fungal community. **(C, D)** represent bacterial community.

Intercropping treatments BKIM, DHIM and CK were compared in pairs. It could be seen from **Figure 3** that the total number of fungal and bacterial OTUs of BKIM was 768 and 2582 respectively, and the number of OTUs shared with BK was 145 and 315 respectively, which increased by 44.78% and 119.03% respectively compared with BK. The total number of fungal and bacterial OTUs of DHIM was 803 and 3047, respectively, and the number of OTUs shared with DH was 147 and 545, respectively, which was 14.09% and 22.88% higher than that of DH, respectively. The more the number of OTUs, the more fungi and bacteria contained in the soil.

**Figure 4:**
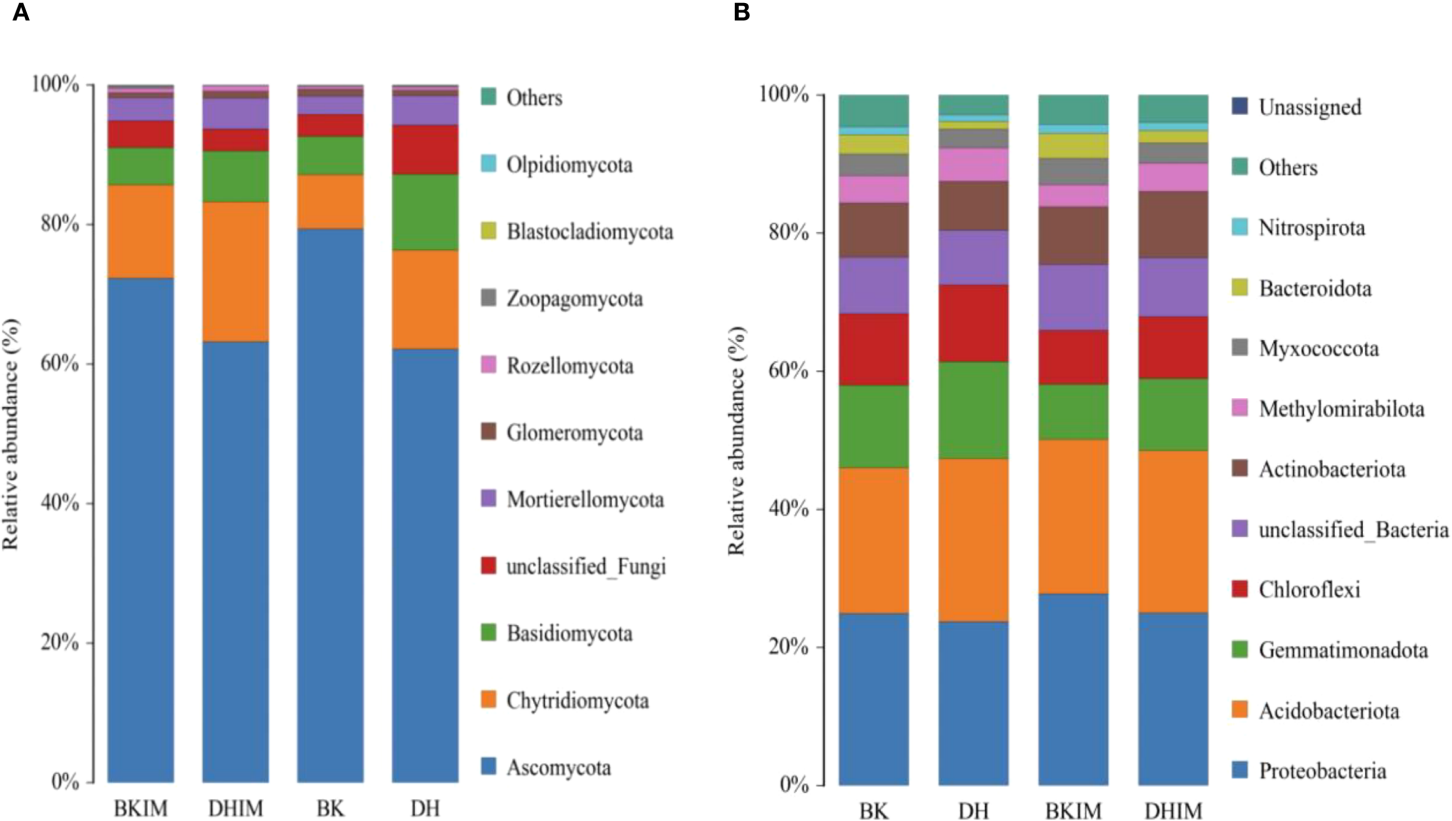
Top 10 fungi and bacteria in the relative abundance of rhizosphere soil under pepper - peanut intercropping mode. **(A)** represents relative abundance of fungi. **(B)** represents Relative abundance of bacteria.

After sequencing analysis, the average fungal community in the soil of pepper and peanut intercropping (BKIM, DHIM) in this study was divided into 11 phyla, 36 classes, 77.5 orders, 150 families, 254 genera, and 306.5 species (**Table 6**), which were higher than monoculture (BK, DH). Ascomycota and Chytridiomycota (relative abundance more than 10%) were the dominant fungal phyla, accounting for about 75% - 85% (**Figure 4A**). In addition, the abundance of *Basidiomycota*, *Mortierellomycota* and *Glomeromycota* was more than 1%. Compared with monocropping, the abundance of *Chytridiomycota* increased by 25% and 81.25% under intercropping conditions (BKIM, DHIM).

**Table 6:** Statistical table of species at all levels under pepper - peanut intercropping mode.

Treatment	Microorganisms	BKIM	BK	DHIM	DH
Phylum	Fungi	11	10	11	10
	Bacteria	32	29	32	28
Class	Fungi	36	29	36	30
	Bacteria	73	67	71	65
Order	Fungi	73	65	82	63
	Bacteria	177	157	175	160
Family	Fungi	144	120	156	133
	Bacteria	288	246	283	252
Genus	Fungi	252	184	256	215
	Bacteria	410	324	412	349
Species	Fungi	293	217	320	265
	Bacteria	440	337	437	367

After sequencing, it was found that the bacterial community in pepper - peanut intercropping soil was divided into 32 phyla, 72 classes, 176 orders, 285 families, 411 genera, and 438.5 species (**Table 6**), which were higher than monoculture (BK, DH). *Proteobacteria* and *Acidobacteriota* (relative abundance more than 10%) were the dominant bacteria, accounting for about 50% (**Figure 4B**). In addition, the abundance of *Gemmatimonadota*, *Chloroflexi* and *Actinobacteria* was more than 1%. Compared with monoculture, the abundance of *Proteobacteria*, *Acidobacteriota* and *Actinobacteria* increased under intercropping conditions (BKIM, DHIM).

As shown in **Table 7**, the OTUs of fungi under intercropping conditions (BKIM, DHIM) increased by 57.05% and 11.22%, respectively, compared with monoculture (BK, DH), and the OTUs of bacteria increased by 91.26% and 18.01%, respectively. Alpha diversity index analysis showed that the richness and diversity index of bacteria and fungi increased after intercropping (BKIM, DHIM) compared with monoculture, and the ACE index and Chao1 index were significantly different.

**Table 7:** Changes of microbial diversity index in rhizosphere soil under pepper - peanut intercropping pattern.

Treatment	Microorganisms	BKIM	BK	DHIM	DH
OTUs	Fungi	768b	489d	803a	722c
	Bacteria	2582b	1350c	3047a	2582b
ACE	Fungi	806.28a	531.60c	829.06a	744.18b
	Bacteria	2610.10b	1369.92c	3068.28a	2600.90b
Chao1	Fungi	787.41b	521.50d	812.64a	729.95c
	Bacteria	2584.72b	1353.08c	3048.26a	2583.44b
Simpson	Fungi	0.9244b	0.9072b	0.9432a	0.9441a
	Bacteria	0.9985a	0.9974a	0.9986a	0.9982a
Shannon	Fungi	5.75a	5.27a	6.03a	5.85a
	Bacteria	10.24a	9.41a	10.42a	10.13a

Different lowercase letters represent significant difference level (*P* < 0.05).

## 4 Discussions

### 4.1 Physiological mechanism of intercropping promoting pepper growth

Multiple factors affect Capsicum spp yield, among which cropping systems constitute a significant determinant. Sorghum - peanut intercropping significantly enhances crop dry matter accumulation (Qiu et al., 2024), maize - peanut intercropping substantially increases maize yield through improved photosynthetic rates (Chen et al., 2023). In this trial, the dry matter accumulation and yield of pepper under pepper - peanut intercropping mode were significantly higher than those under monoculture, indicating that intercropping with peanut could effectively improve the agronomic traits, dry matter accumulation and yield of continuous cropping pepper, which was related to the additional nitrogen source provided by peanut nitrogen fixation (Betencourt et al., 2012). This is basically consistent with the results of previous studies. It shows that the intercropping mode has a certain universality in promoting the dry matter accumulation and yield of crops.

In this study, the photosynthetic rate and transpiration rate of intercropped pepper were significantly higher than those of monoculture at the full fruit stage, probable due to intercropping creates a more favorable microclimate environment for farmland, optimizes the utilization efficiency of resources (light, water, nutrients), and alleviates environmental stress through interspecific interactions (Brooker et al., 2015; Wang et al., 2019). In addition, the peanut canopy might improve the field microclimate and alleviate the photoinhibition of pepper (Gao et al., 2010). As a leguminous plant, peanut has the ability of symbiotic nitrogen fixation, which can improve the supply of soil nitrogen, thus promoting the photosynthesis and growth of pepper (Xiao, 2003a; Chen et al., 2023). Future research can further explore the cycle and utilization efficiency of nitrogen in pepper/peanut intercropping system.

### 4.2 Dynamic response of soil nutrients and enzyme activity

Qiu et al. (2024) found that sugarcane - peanut intercropping could significantly increase the content of available phosphorus and organic matter in the soil, which was similar to the results of this study. Under the pepper - peanut intercropping mode, the content of available phosphorus and organic matter in the rhizosphere soil of pepper was higher than that of monoculture, while the content of available potassium decreased. This might be related to the strong absorption capacity of peanut roots to potassium (Tang et al., 2011; Zuo et al., 2000). Organic matter was significantly accumulated at the full fruit stage (DHIM reached 19.92 g kg^-1^), which was attributed to the increase of peanut litter input and root exudates (Vandenkoornhuyse et al., 2015). In addition, the change of soil enzyme activity under intercropping mode showed that the increase of soil invertase and urease activity at maturity stage might be related to the improvement of microbial community structure (Dai et al., 2015), which reflected that intercropping delayed the decline of soil enzyme activity and was beneficial to nitrogen mineralization. Similarly, Qiu et al. (2024) found that sugarcane - peanut intercropping significantly increased soil enzyme activity, further demonstrating the positive impact of intercropping patterns on soil fertility. Intercropping patterns provide a better soil environment for crop growth by improving soil nutrients and enzyme activities.

### 4.3 Key changes in microbial community structure

High-throughput sequencing showed that intercropping significantly increased bacterial - fungal OTUs and α diversity index (**Tables 5**-**7**). Among them, BKIM bacterial OTUs increased by 91.26% compared with BK, and the abundance of Proteobacteria increased. This phylum contained a large number of PGPR, such as *Pseudomonas* and *Rhizobium* (Bulgarelli et al., 2013), which might promote the growth of pepper by secreting growth hormone or antagonizing pathogens. The abundance of *Chytridiomycota* in the fungal community increased by 81.25% (DHIM *vs* DH) under intercropping, and its members could degrade organic phosphorus (Tedersoo et al., 2014), which was consistent with the increase of available phosphorus content. In addition, this study found that intercropping significantly improved the soil microbial community structure, and Liu et al. (2020) also obtained similar results under maize-peanut intercropping. However, the mechanism of microbial community structure changes on plant disease prevention and control needs to be further studied.

Pepper - peanut intercropping synergistically through “nutrient complementation-microorganism interaction”, but it should be noted that peanut might compete for potassium and lead to a decrease in available potassium at maturity. Intercropping had no significant improvement effect on soil pH, and alkaline soil still needed to be regulated. In the future, alkali-tolerant pepper varieties can be screened or potassium-solubilizing bacteria can be introduced to optimize the model.

## 5 Conclusions

The pepper - peanut intercropping model significantly increased the yield of processed pepper and the quality of soil microenvironment. The main conclusions were as follows. The yield per mu of “Beike 802” and “Dehong 1” increased by 15.01% and 9.12%, respectively, the dry matter accumulation increased by 17.94% -25.46%, and the photosynthetic rate (Pn) increased by 47.93% -59.06% in the full fruit period. The soil organic matter content reached 19.92 g kg^-1^, the available phosphorus increased by 9.99% -12.5%, but the available potassium decreased by 12.3% -29.4%. The activities of urease and sucrase in mature stage were significantly higher than those in monoculture. Intercropping significantly increased bacterial and fungal OTUs (the highest increase was 91.26%), ACE and Chao1 diversity index increased. The abundance of *Proteobacteria* and *Chytridiomycota* increased, forming a more stable microbial network. This model alleviated pepper continuous cropping obstacles by enhancing resource utilization efficiency and microbial interaction. It was recommended to promote the application in the pepper area of the Huang-Huai-Hai Plain, and further study the potassium regulation strategy.

## Data Availability

The original contributions presented in the study are included in the article/**Supplementary Material**. Further inquiries can be directed to the corresponding author.
