# Supplementary material for: Effects of pepper – peanut intercropping systems on processed chili yield and rhizospheric soil microecological environment
Source: Front Plant Sci. 2025 Oct 14;16:1666686. doi: 10.3389/fpls.2025.1666686 (PMC12558943; doi:10.3389/fpls.2025.1666686)
Supplement: Supplementary file 1 [file DataSheet1.docx]

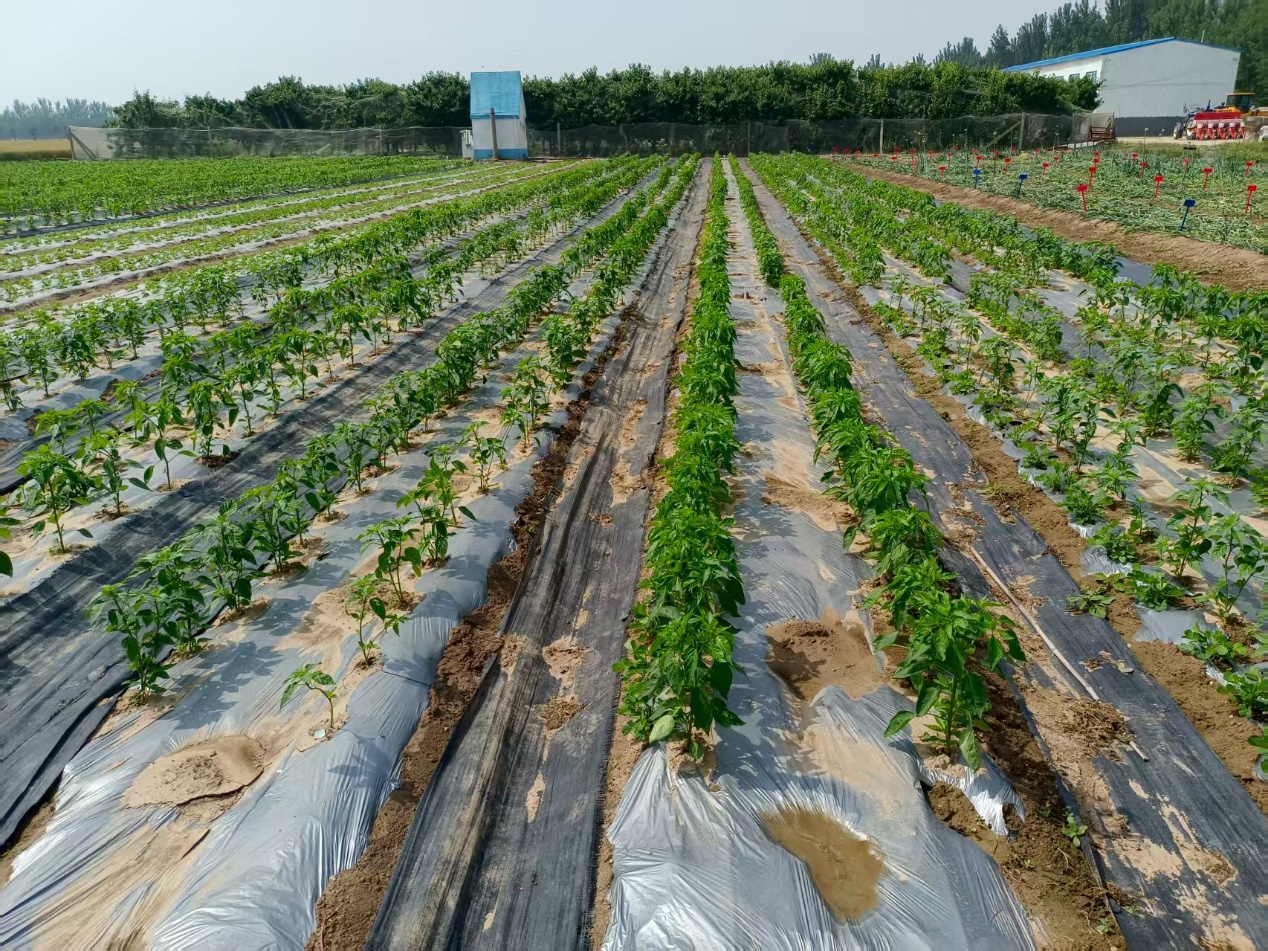


Schematic diagram of intercropping between chili peppers and peanuts


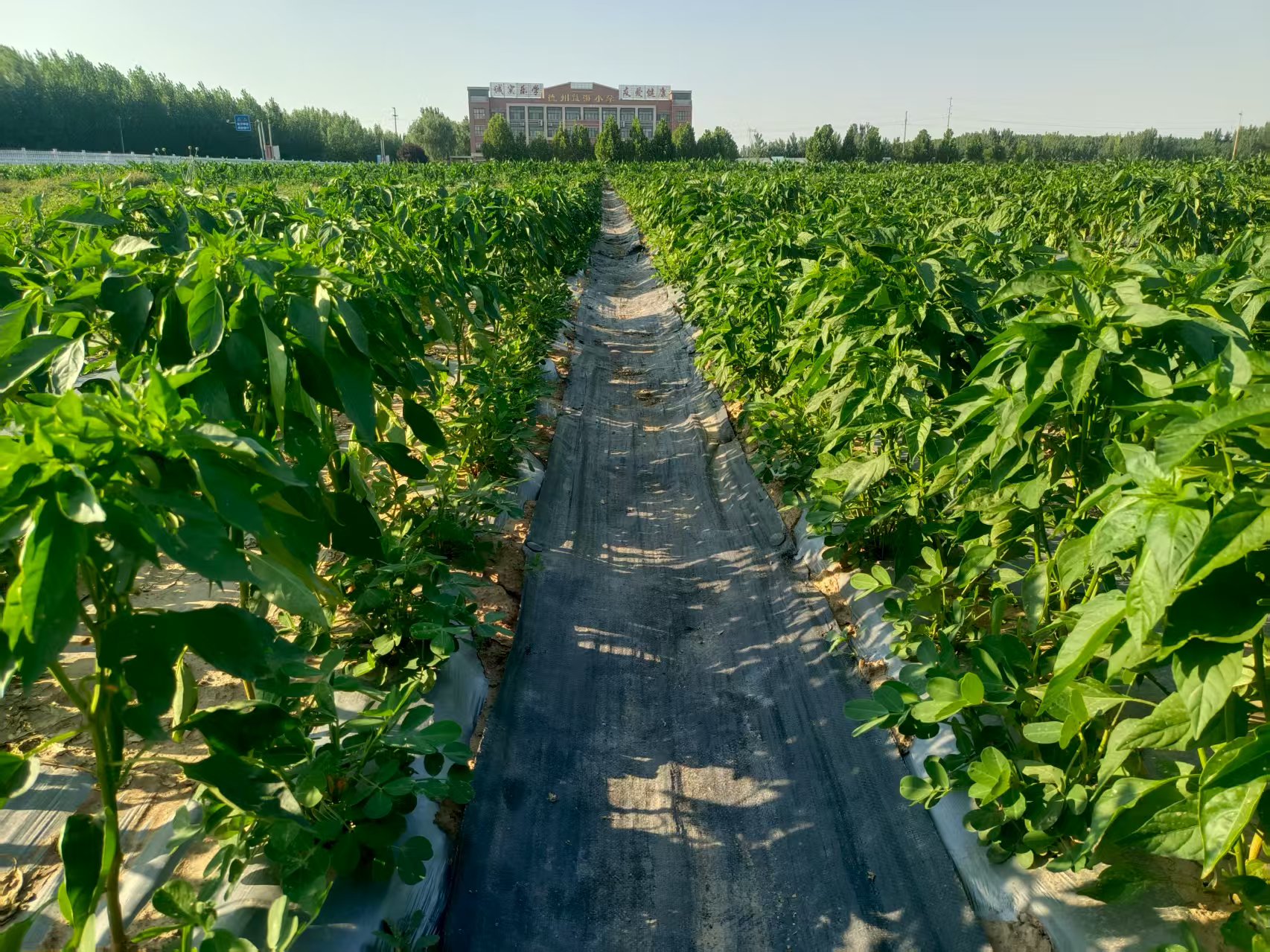


Schematic diagram of intercropping between chili peppers and peanuts
